# Olfaction and gustation in blindness: a state of the art of the literature

**DOI:** 10.1007/s10072-023-06734-8

**Published:** 2023-03-13

**Authors:** Maria Jimena Ricatti, Silvia Savazzi, Paola Cesari, Maria Paola Cecchini

**Affiliations:** 1 Department of Neurosciences, Biomedicine and Movement Sciences, Anatomy and Histology Section, University of Verona, Strada le Grazie 8, 37134 Verona, Italy; 2 Perception and Awareness (PandA) Laboratory, Department of Neuroscience, Biomedicine and Movement Sciences, University of Verona, Strada le Grazie 8, 37134 Verona, Italy; 3 Department of Neurosciences, Biomedicine and Movement Sciences, Movement Sciences Section, University of Verona, Via Casorati 43, 37131 Verona, Italy

**Keywords:** Blindness, Olfaction, Gustation

## Abstract

To date, there are quite a few studies assessing olfaction and gustation in blindness, with great variability in sample size, participants’ age, blindness onset and smell and taste evaluation methods. Indeed, the evaluation of olfactory and gustatory performance can differ depending on several factors, including cultural differences. Therefore, here we analysed through a narrative review, all the works reporting a smell and taste assessment in blind individuals during the last 130 years, trying to summarize and address the knowledge in this field.

## Introduction

Evolutionarily, smell and taste are the oldest of our senses, greatly differing when compared to the younger senses as vision and audition. Interestingly though, vision is the most powerful sense to interact with the environment and for a very long time, such that it has been hypothesized that individuals affected by congenital blindness develop better abilities in making use of the other senses due to a supposed compensatory plasticity of the visual brain areas in the processing of non-visual information. While numerous studies investigated auditory and/or tactile functions in early blind individuals [1, 2], only few evaluated olfactory and gustatory functions, so these abilities in blind participants are not fully clarified so far. Considering these premises, here we provide a review of the state of the art about olfaction and gustation in blind individuals with the purpose to address the current knowledge in this field. Moreover, to provide a better understanding of the available results, we also consider the various evaluation methods to guide readers in the understanding of limits and strengths of the available literature over the years.

## Methods

A literature search was performed in English on PubMed and Scopus electronic databases. The search in PubMed for articles was carried out by using 2 sets of terms combination. The first set included (“Olfaction” [MeSH] OR “Olfactory performance” [MeSH] OR “Olfactory perception” [MeSH]) AND (“Gustation” [MeSH]) AND (“Blind” [MeSH] OR “Blindness” [MeSH]) and the second set of terms (“Smell” [MeSH] OR “Smell perception” [MeSH]) AND (“Taste” [MeSH] OR “Taste perception”) AND (“Blind” [MeSH] OR “Blindness” [MeSH]).

The advanced Scopus search used the terms (“Olfaction” OR “Olfactory perception” OR “Olfactory performance”) AND (“Gustation”) AND (“Blind” OR “Blindness”). The inclusion criteria covered peer-reviewed articles (languages: English, Italian, German, French). The research included papers involving blind participants, where olfactory and gustatory performance was measured by means of specific sensory tests. Reviews and studies that used only self-assessment questionnaires and/or articles in which there were only blind-folded participants or sighted in the dark subjects, were excluded. A narrative review approach was chosen due to the emerged methodology differences not easy to compare. The selected papers are summarized in Table 1.

**Table 1:** Papers assessing olfaction and/or gustation in blindness included in the review

Authors, Year	Population	Results
Griesbach (1899)	20 blind 40 sighted	Olfaction No differences between blind and sighted participants
Mahner (1909)	4 blind 4 deaf-mute 4 sighted (8–14 years)	Olfaction and gustation Blind participants are superior to the other two groups
Bertoloni (1942)	20 blind (congenitally or present for more than 6 years)	Olfaction Enhanced olfactory function in all blind individuals (congenital or not)
Cherubino & Salis (1957)	31 blind (age: 5–35) 55 deaf-mute 86 sighted (10–15 years)	Olfaction No evidence of enhanced olfactory sensitivity in blind persons
Boccuzzi (1962)	31 congenitally blind 65 blind for > 7 years 100 sighted	Olfaction No overall differences are observed between the sighted and blind participants (congenital or not)
Freund (1973)	3 blind males 2 blind females (20–45 years)	Olfaction and Gustation in food experience (coffee powder) Blind participants can be trained for coffee evaluation
Murphy & Cain (1986)	20 early and late blind (21–68 years) 20 sighted (20–67 years)	Olfaction Blind participants have lower sensitivity threshold than the sighted, while they perform better on the odour identification test
Terner et al. (1987)	46 sighted dyspeptic (20–70 years) 42 blind individuals (22–70 years)	Gustation 16% of the blind participants showed strong hypergeusia when the taste thresholds were lower, three times more frequent than the dyspeptic group. PTC (Phenylthiocarbamide) bitter sensitivity was found to be equal in both groups tested
Smith et al. (1993)	31 congenitally blind 18 blind for more than 10 years 7 blind for less than 10 years (29–54 years) 68 sighted (27–49 years) 23 sighted from a panel (29–51 years)	Olfaction and Gustation Blind participants do not significantly outperform their untrained sighted counterparts on any olfactory tests Taste identification test shows that trained sighted participants score higher than the other groups
Rosenbluth et al. (2000)	27 congenitally blind 3 blind since age of 3 30 sighted (5–17 years)	Olfaction No increase in olfactory proficiency among blind children, in terms of olfactory threshold, forced-choice or odour-labelling scores
Schwenn et al. (2002)	15 blind (congenitally and non-congenitally) 15 sighted (21–51 years)	Olfaction Neither with subjective nor with objective methods differences were found
Wakefield et al. (2004)	32 early blind 5 late blind 14 low vision 32 sighted (8–18 years)	Olfaction Early blind children outperform on the odour-naming task but not on the odour-sensitivity task
Cuevas et al. (2009)	13 blind before age of 2 (23–47 years) 13 sighted (23–47 years)	Olfaction Blind participants significantly outperform in free-identification of odors and, to a slightly less extent, in odour discrimination and categorization
Rombaux et al. (2010)	10 blind before age of 2 (23–57 years) 10 sighted (22–55 years)	Olfaction Blind participants have larger olfactory bulb volume by MRI scan The free-identification and odour discrimination tasks yield higher scores in blind participants
Beaulieu Lefebvre et al. (2011)	11 congenitally blind (18–46 years) 14 sighted (21–39 years)	Olfaction Blind participants have a significantly better odour detection threshold but do not outperform in terms of odour discrimination and identification
Kupers et al. (2011a)	11 congenitally blind (18–46 years) 14 sighted (21–39 years)	Olfaction Congenitally blind participants more strongly activate olfactory areas during odour detection task and odour condition
Gagnon et al. (2013)	11 congenitally blind (32–40 years) 13 sighted (31–37 years)	Gustation Blind participants have a reduced taste sensitivity in terms of detection and identification thresholds
Luers et al. (2013)	19 congenitally blind 27 severe bilateral visual impaired (18–55 years) 46 sighted (matched pairs with maximum age difference 3 years)	Olfaction No significant difference between groups. Control group shows a significant difference in the average discrimination score
Gagnon et al. (2015)	9 congenitally blind (40–50 years) 14 sighted (35–43 years)	Gustation Blind participants show weaker taste-induced responses
Comoglu et al. (2015)	17 congenitally blind 16 acquired blind (25–45 years) 33 sighted (18–49 years)	Olfaction Blind participants appear to be better in odour threshold, discrimination and TDI total scores
Gagnon et al. (2015)	12 congenitally blind (38–46 years) 12 sighted (36–44 years)	Olfaction Congenitally blind participants are better and faster in identifying odorants presented via the orthonasal but not via the retronasal route
Kärnekull et al. (2016)	30 blind 30 sighted individuals (sex and age matched) (24–74 years)	Olfaction No evidence of an overall superior performance in blind individuals. For odour discrimination and identification, there are relatively modest differences between early blind, late blind and controls
Sorokowska (2016)	43 early-blind 41 late-blind 84 sighted (matched pairs with maximum age difference 3 years) (16–65 years)	Olfaction No significant effect of “sight” on any Sniffin’ Sticks subtest, free identification score or the total TDI score
Manescu et al. (2018)	12 early-blind 12 sighted (24–71 years)	Olfaction in food experience (wine) Early-blind individuals found it hard in wine odour categorization No differences in odour differentiation and classification
Sorokowska et al. (2020)	51 early-blind (23–41 years) 49 late-blind (30–50 years) 99 sighted (21–41 years) 74 deaf individuals (19–42 years) 100 hearing controls (21–41 years)	Olfaction Early blind participants detect fish odour in significantly lower concentrations than late blind participants
Manescu et al. (2021)	10 congenitally blind (21–62 years) 10 sighted (23–58 years) 10 late-blind (38–66 years) 10 sighted (37–63 years)	Olfaction Congenitally blind participants but not late-blind late show enhanced localization of chemosensory stimuli across nostrils, most probably of the trigeminal component

## Results

Considering the last 130 years, there are twenty-six studies reporting olfaction and/or gustation assessment in blind individuals, and most of them investigated only olfactory sensitivity threshold. In addition, the results reported are rather contradictory, often obtained by testing few individuals, or considering different methods of analysis on both congenital and/or adult-onset blindness, including cases where the causes of blindness were obscure. Olfactory and gustatory assessment methods are summarized in Table 2.

**Table 2:** Olfaction and gustation assessment methods reported in the studies involving blind individuals

Authors	Methods Olfaction	Description
Griesbach (1899)	Zwaardemaker’s drawtube olfactometer	This is a device developed in 1888 by the Dutch physiologist Hendrick Zwaardemaker for determining olfactory thresholds, and contains a tube that pushes a fixed amount of a selected gaseous odorant into the nose of the patient
Mahner (1909)	Puff delivery	Olfactory discrimination between two different-sized puffs of two stimuli: Lily of the Valley perfume and a sulphur-smelling agent. Taste response to two different-sized puffs of each of three stimuli (chloroform, ether, and a 40% solution of acetic acid)
Bertolini (1942)	Elsberg blast-injection	The absolute threshold is determined by finding the magnitude of blast of odorous air injected into the participants’ nostrils which will give rise to odour perception. This is accomplished by the very simple device of releasing, through a pinchcock, a known amount of odorous air which has been compressed by means of a hypodermic syringe
Cherubino & Salis (1957) Boccuzzi (1962)	Fortunato & Niccolini’s olfactometer	This olfactometer consisted of six bottles with 530 ml in which 30 ml of the odorous substances are placed (anethol, phenyl–ethyl-alcohol, citral, menthol, vanillin, and pyridine) plus 500 ml of air. First, 5 ml of the odorous air is insufflated into the participant’s nose; if the participant is not able to detect or differentiate the odour, the amount of air (10, 15, 20 ml) is increased in order to define the thresholds of perception and identification for a particular odorous substance
Murphy & Cain (1986)	Identification of everyday odours	80 Common odorants such as baby powder, mothball and cigarette butts are presented in random order in small jars held under the nostril of blindfolded participants
Murphy & Cain (1986) Rosenbluth et al. (2000) Wakefield et al. (2004)	Threshold test	Detection in a single ascending series threshold task by smelling n-butyl-alcohol from squeezable bottles. The odorant is prepared in a series beginning with 4% v/v in deionized water and each successive dilution is one-third the concentration of the preceding dilution. participants are presented with one target bottle and one blank bottle in succession for recognizing the target smell by squeezing the bottle once in a randomized order across trials
Smith et al. (1993)	Identification test (UPSIT, Sensonics, USA)	A participant is required to identify, in a four-alternative multiple-choice format, each of 40 odorants presented on microencapsulated “scratch and sniff” labels
	Threshold test	This test has the rose-smelling odorant PEA diluted in mineral oil in a concentration series range (v/v). Test starts with the presentation of two sniff bottles, containing a given PEA concentration or the diluent alone in rapid succession and in random order. Participants are required to report which of the two bottles contains the strongest smell, and if no difference is perceived, a forced choice is required
	Discrimination test	This test determines whether an individual can discriminate qualitative differences among odorants independent of the ability to recognize their name. The participant is presented with 16 sets of three microencapsulated odorants in which two are the same and one is different and is asked to select the different odour within each triad. The number of correctly answered items serves as the test score
Rosenbluth et al. (2000)	Free-choice labelling	This is a supra-threshold test by discrimination of odours, requiring the identification (labelling) of 25 common items to test for proficiency at discriminating/generating labels, smelling one by one in a random order
Rosenbluth et al. (2000) Wakefield et al. (2004)	Forced-choice labelling	The test involves selecting the correct label of each odour stimulus from a list of four labels provided (verbally) after each trial
Schwenn et al. (2002)	Olfactory and trigeminal evoked potentials	Vanillin was used for assessing the olfactory evoked potentials while carbon dioxide and hydrogen sulfide for trigeminal evoked potentials
Wakefield et al. (2004)	Paired-associate memory	The test is presented as an odour-word game involving naming new perfumes, where participants must pair an unfamiliar odour with a provided label
Cuevas et al. (2009) Rombaux et al. (2010)	Discrimination (sentosphere)	Each trial consisted in a presentation of a pair of odours constituted pseudo-randomly using a set of 30 commercially available bottles that contained microencapsulated granules of odorants selected by a perfumer and when opened diffused a fragrance One-half of the pairs contained the same odorant, whereas the others contained a different one. Participants are required to smell each stimulus of the pairs and to determine whether the second odour is the same or different with respect to the first one
	Identification (sentosphere)	The test consists in a presentation of 30 commercially available bottles that contain microencapsulated granules of odorants presented one by one Participants are required to smell the odorant and to name it (free-identification) Then, each stimulus is presented a second time to categorize the stimulus (fruit, flower, plant or other). Lastly, participants are required to identify the stimulus by selecting its name by multiple choice identification
Schwenn et al. (2002) Beaulieu Lefebvre et al. (2011) Luers et al. (2013) Comoglu et al. (2015) Sorokowska (2016) Kärnekull et al. (2016)	Threshold, discrimination and identification (Sniffin’ Sticks Extended test, Burghart, Germany)	Threshold is assessed by using 48 pens in a single staircase method in ascending concentration. Odour discrimination is evaluated by presenting 16 triplets of odorants, of which two are the same and one is different. For odour identification, 16 pens with common smells (for example orange, cinnamon, onion, banana, lemon or fish) are presented and participants had to identify the odour by selecting one of four possible descriptors. The scores of each test could vary between 0 and 16, and are summed into a total TDI (Threshold, Discrimination and Identification) score. Higher scores indicate better performance
Kupers et al. (2011a)	Detection	Solutions made with phenylethyl alcohol 5% (v/v) in propanediol and butanol 5% (v/v) in propanediol, in both cases detection thresholds are known to be the same, delivered via a computer-controlled olfactometer during fMRI scanning
Gagnon et al. (2015)	Identification (Free-choice and forced-choice)	This test uses 16 grocery store condiments as olfactory stimuli by placing a plastic vial containing the food powder 5 cm below the participant’s nose. The participant is asked to take two normal breaths and identify as fast as possible the odorant. Additionally, the test is administered again with 4 verbal labels to choose from
	Identification (Free-choice, retronasal)	This test uses 16 grocery store condiments as olfactory stimuli by placing the grocery powder on the tongue using a teaspoon, while the participant had the nostrils closed. After stimulus delivery, the participant is asked to close the mouth, open the nostrils, breathe normally and identify the odorant
Sorokowska et al. (2020)	Threshold	The test uses fish sauce to produce the odour of a rotten fish, which is edible, unpleasant and its odour is associated with food decay relevant for food-related hazard detection. The threshold test mirrored a popular Sniffin’ Sticks threshold wherein the participants are presented with triplets of pen-like dispensers, two unscented and one odorized
Manescu et al. (2021)	Odorant localization	Olfactory and trigeminal stimuli (i.e. almond and eucalyptus odours) are delivered with an adapted computer-controlled air compressor system and the burning/tingling/cooling sensations and detected
	Detection	Odorants related to edible food (i.e. strawberry and parmesan cheese aroma) are delivered with an adapted computer-controlled air compressor system and participants are requested to identify the stimuli
Authors	Methods Gustation	Description
Maher (1909)	Puff delivery	Taste response to two different sized puffs of each of three stimuli (chloroform, ether, and a 40% solution of acetic acid)
Terner et al. (1987)	Detection and recognition threshold	Forced-choice 3 gustatory stimuli drop technique for the four taste modalities (sucrose, sodium chloride, citric acid, urea) The detection threshold here referred is the lowest concentration of the test solution that the subject can consistently distinguish from distilled water The recognition threshold is the lowest concentration of the test solution which the participant can identify as a tastant
	Bitter sensitivity test	PTC (phenylthiocarbamide) for bitter sensitivity assessment
Smith et al. (1993)	Whole-mouth taste test	This method is based on a suprathreshold whole-mouth taste identification, intensity, and pleasantness test. Five ascending concentrations of sucrose, citric acid, sodium chloride and caffeine are tasted by a participant in counterbalanced order, with distilled/deionized water rinsings interspersed between presentations. Each stimulus is presented twice, resulting in a total of 40 trials per participant
Gagnon et al. (2013)	Threshold and identification	Detection and identification taste threshold of 5 series sucrose, NaCl, citric acid, quinine and MSG. Tastants are randomly presented following the 2-alternative forced choice “sip and spit” fashion
	Bitterness sensitivity (Fischer Scientific)	PTC for bitterness sensitivity assessment using taste strips
Gagnon et al. (2015)	Gustometer	Sucrose and quinine hydrochloride in two different concentrations (weak and strong), plus artificial saliva are manually delivered by using the gustometer during fMRI scanning session. Participants are asked to swallow all liquids during the scanning sessions

### Works on olfaction

Since the beginning of the nineteenth century until today, there are few publications assessing olfactory performance in blind individuals. Beyond the preliminary works on olfactory threshold through pioneering olfactometers [3–6], Murphy and Cain in 1986 were the first to examine olfactory identification ability in 20 blind adults, including both early blind and late blind. Participants were assessed by means of a threshold and an identification task, with everyday odours homemade prepared. The identification test consisted of two sessions separated by a period of three days and the test was based on a free-choice method (without choice options, more difficult task). Participants were informed about the veridical odour name when named incorrectly. Results showed that sighted persons had significantly better sensitivity, but they were poor in performing the identification task, while the blind could name 31% more odours than the sighted individuals [7].

In 2000, Rosenbluth and co-workers compared the performance of 30 congenital blind children (age range: 5–17 years) with that of 30 normal sighted controls. They were tested both for olfactory threshold and olfactory identification task with 25 common odours in both a free-choice and forced-choice assessment. Early blind children were better and faster in a free-choice identification, but there was no increase in overall olfactory proficiency among blind children [8]. In 2002, Schwenn and collaborators investigated whether blind persons have a stronger sense of smell. They studied the olfactory function in 15 participants, both congenitally and non-congenitally blind, plus 15 sighted controls. Sniffin’ Sticks Extended test was performed in order to assess participants olfactory threshold, discrimination and identification. In addition, olfactory and trigeminal evoked potentials were also analysed, but no differences were found with any of the methodologies used [9]. Wakefield et al. in 2004 extended the Rosenbluth research by examining the role of cognitive factors in blind children on an odour-naming task. They found that blind children (*n* = 32) outperformed the normal sighted ones (*n* = 32). Blind children showed a particular advantage on tasks assessing memory for non-visualizable stimuli [10]. Then, Cuevas et al. in 2009 studied 13 early blind and 13 normal sighted participants. They evaluated odour discrimination ability and odour identification by means of 30 pairs of odours (www.sentosphere.fr). The identification task was performed first in a free-choice format, and then each stimulus was presented a second and a third time when the participant was asked to categorize it by choosing among different semantic categories (forced-choice assessment). Results showed that blind participants significantly outperformed the normal sighted controls in odour discrimination, free-choice identification and categorization of odours. For the forced-choice identification, blind individuals performed slightly better than normal sighted, but the difference was not significant [11].

In 2010, Rombaux et al., in addition to olfactory function in early blind participants, also measured olfactory bulb volume by magnetic resonance imaging (MRI). They studied 10 early blind participants and 10 normal sighted controls. Olfactory function was assessed by applying discrimination and odour free-choice identification tasks by means of a set of 30 selected commercially available odorants (www.sentosphere.fr). Results showed that early blind participants were superior to controls in both the aforementioned olfactory tasks and the blind’s olfactory bulb volume (right and left) was higher in the blind compared to the control group [12]. Beaulieu-Lefebvre in 2011 assessed 11 congenital blind participants and 14 normal sighted controls by means of the Sniffin’ Sticks Extended test, a validated olfactory test battery that comprises three subtests: threshold, discrimination and identification. All tasks requested were forced choice. In addition, they administered a questionnaire to evaluate the self-reported odour awareness by means of the Odour Awareness Scale (OAS). Results showed that the blind group had a significantly better odour threshold (lower) in comparison with the normal sighted controls. The other performances were not different. Moreover, by means of the awareness questionnaire, it interestingly appeared that blind participants were more aware of their olfactory environment [13].

Thereafter, Kupers and collaborators in 2011 investigated how the olfactory system is influenced by the lack of vision in congenital blindness by means of the functional magnetic resonance imaging technique (fMRI). They assessed 11 congenital blind participants and 14 normal sighted controls. Results showed that blind people process olfactory stimuli differently. In particular, compared to normal sighted controls, congenitally blind participants, beyond a strong activation in the piriform cortex and orbitofrontal cortex, had significantly larger response at the level of the overall occipital cortex. This represents the first study demonstrating an involvement of the blind’s visual cortex in olfactory processing [14]. In 2014, Luers and co-workers evaluated olfactory performance on 46 blind and 46 normal sighted participants respectively, by means of the Sniffin’ Sticks Extended test (Burghart, Germany) assessing threshold, discrimination and identification domains showing no significant differences between the groups [15].

Later, Gagnon et al. in 2015 investigated both orthonasal and retronasal olfactory perception on a sample of 12 congenital blind people and 14 normal sighted controls [16]. They used 38 grocery powders as olfactory stimuli, 19 for orthonasal and 19 for retronasal stimulation, according to the protocol of Heilman and co-workers [17]. For both tests, after the stimulus delivery, the participant was asked to identify it in a free-choice assessment, then with a forced-choice selection. Results revealed that congenital blind participants were better than controls in identification of odorants via orthonasal route. Moreover, they had a better performance also in the free-identification format.

In the same year, Comoglu et al. assessed the sense of smell in 33 blind participants (17 congenital and 16 acquired) and 33 normal sighted controls by means of the Sniffin’ Sticks Extended test as in the previous work of Luers et al. (2014) [18]. Results showed a better performance of the blind individuals globally compared to normal sighted controls, while no difference was found between congenital and acquired blindness. Olfactory threshold was found to be better (perception at lower concentrations) in the blind group, and blind participants significantly outperformed normal sighted controls also in olfactory discrimination test as well as considering the total score, sum of the three subtests.

In 2016, Sorokowska et al. enrolled a large sample size of participants (43 early-blind, 41 late-blind and 84 sighted participants) and evaluated olfactory performance through the Sniffin’ Sticks Extended test showing no difference among groups [19]. Same year, Kärnekull et al. showed no evidence of an overall superior performance in blind relative sighted individuals by Sniffin’ Sticks Extended test (15 early-blind, 15 late-blind and 30 sighted controls) [20].

A later study from Sorokowska et al. in 2020 (49 late-blind, 51 early-blind, 100 sighted controls) reported no evidence of olfactory compensation by using a custom-made threshold for odour detection test with a fish sauce odour [21].

More recently, Manescu and collaborators assessed odorant localization and detection delivered with an adapted computer-controlled air compressor. They observed that congenitally blind (10 participants) outperformed sighted (20 participants) and late-blind (10 participants) in a birhinal localization task by means of mixed olfactory and trigeminal stimuli. On the other hand, they did not observe any blind advantage for odorant detection or identification, suggesting that the trigeminal component might have a different processing in the congenitally blind individuals [22].

### Works on gustation

To our knowledge, only three studies assessed gustatory performance alone. First, Terner and collaborators in 1987 [23] investigated gustation for the four basic taste modalities (sweet, sour, salty, bitter) in 46 dyspeptic patients and 42 blind participants as the control group, by means of the three stimuli drop technique [24, 25]. In addition, PTC (phenylthiocarbamide) bitter sensitivity was measured. They found that the 16% of the blind participants showed strong hypergeusia when the taste thresholds were lower, three times more frequent than the dyspeptic group. Moreover, neither hypogeusia nor ageusia was found in both two examined groups and PTC sensitivity was found to be equal in both groups tested [23]. Despite that, it is meaningful to highlight that in this work the authors did not include a healthy control group. Later in 2013, Gagnon and collaborators [26] measured taste detection and identification thresholds of the five basic tastes including umami, the fifth taste, determined by L-glutamate, amino acids and purine nucleotides [27]. The sample population was made of 11 congenitally blind and 13 normal sighed controls. Results showed that blind participants have a reduced taste sensitivity in comparison with the normal sighted controls (higher taste detection and identification thresholds). Interestingly, blind participants were better in the umami identification threshold. Then, in 2015, Gagnon and co-workers evaluated taste perception in congenital blindness, by means of functional magnetic resonance imaging (fMRI) [28]. In particular, they assessed 20 congenital blind individuals and 14 normal sighted controls with different taste solutions of the 4 basic tastes (sweet, sour, salty, bitter) as well as with artificial saliva administration. Results showed that, compared to the artificial saliva stimulation, taste solutions evoked a less strong signal into the primary gustatory cortex and hypothalamus. In addition, differently from the studies assessing other sensory modalities such as audition, touch and olfaction, the occipital cortex was not activated during taste stimulation in the blind participants. The authors suggested that during gustatory processing there is no compensatory mechanism taste-related, involving the occipital cortex.

### Works on both olfaction and gustation

Two studies focused on both olfactory and gustatory assessment in blind participants. Mahner published the first old work in 1909 with only 4 participants per experimental group enrolled, including blind, deaf-mute and sighted participants. According to his results, blind individuals were superior in terms of olfactory discrimination. Taste evaluation showed that blind participants were better in making taste discrimination. However, these results were questionable, providing tactile cues related to pressure, volume and duration of stimulation. Moreover, several of the stimuli were likely able to stimulate the trigeminal, as well as the olfactory or gustatory system [29].

In 1993, Smith and collaborators evaluated chemosensory function in a group of 56 blind participants and 91 controls. The pool of blind people was composed by 31 congenital blindness, 7 adult-onset blindness (< 10 years) and 18 adult onset blindness (˃10 years). Interestingly, among the 91 sighted controls, there was a panel of 23 employers of the Philadelphia Water Department, trained members of a water quality evaluation panel. Olfaction was assessed by means of the UPSIT smell identification test (University of Pennsylvania Smell Identification Test, Sensonics, USA) in four alternative choice options, with 40 odours microencapsulated in “scratch and sniff” labels. In addition, a detection threshold and a discrimination test were administered. Taste was evaluated by a supra-threshold whole mouth taste identification, intensity and pleasantness with five concentrations of the four basic tastes. The authors conclude that blind participants did not outperform normal sighted on basic chemosensory tests and the trained participants scored higher than the other groups in all tests [30].

### Works on food experience and blindness

To our knowledge, two works reported sensory food experience in blind individuals [31, 32]. The first study reported an old rudimental sensory panel blind experience by coffee powder tasting, designed to boost the employment of blind personnel in the food industry. The second more recent study assessed whether a group of early-blind individuals show different smell performances during a pure wine odour evaluation compared to sighted controls, all blindfolded. The authors showed that early-blind individuals found it hard in wine odour categorization (i.e. if both presented wine pairs belonged to the same category, red, white, rosè). On the other hand, no differences with the sighted individuals emerged on the other tasks: odour differentiation (i.e. to indicate if the wines were identical or not) and odour classification (i.e. to classify the wines into red, white and rosè).

Concerning this topic, we would like to report our preliminary experience as an “on the fringes” event of the Neuroscience Congress in Milan, named “Tasting in the dark” which took place at Villa della Torre, Verona, Italy, in 2014. This event involved three groups of people: congress participants (non-expert/normal sighted, *n* = 28), experts (wine producers/sommeliers, *n* = 17) and late onset blind participants (non-expert, visually impaired/blind, *n* = 8).

Participants had to taste six different brands of Amarone Valpolicella wine, of the same year of production (2009) and all of them (normal sighted and blind) were blindfolded. Each wine was assessed following a questionnaire having five items: smell (red fruits, cherries), taste (bitter), astringent, persistent and pleasant. Each item was evaluated following a VAS from 0 (not at all) to 10 (extremely). Another question was to associate the wine with an emotion (no emotion, tension, relaxation, happiness). Few participants underwent an additional chemosensory test (the “Sniffin’Sticks Extended test” for smell evaluation and “Taste Strips Test” for taste evaluation, Burghart, Germany): 8 from the group of congress participants, 10 from the expert group and 8 blind group. In addition, during the identification test (16 different odours), the participant was asked to identify each odour before and after the presentation of four written options per odour each time. From this pilot experience, we found a significant interaction between wine descriptors X group, *p* = 0.013, and the multiple comparisons showed, particularly for the blind group, the ability to discriminate among the descriptors of the different wines. Within the olfactory and taste *status*, we found one close to be significant correlation (*p* = 0.056) only for blind people, considering the test for smell identification without options compared to the one with options (data not published).

## Discussion and future directions

From the reviewed literature, it clearly appears that regarding olfaction there are different studies in blind participants, but works are often performed on small samples, by applying different methods, sometimes not standardized or validated so not easy to compare. Another limit that appears is that the blind population evaluated was heterogeneous, including individuals with congenital and/or acquired blindness and background delivering clinical information sometimes not satisfactory.

However, an important “message” coming out from these interesting papers is that the blind participants generally do not outperform normal sighted controls when using basic tests, while they perform better when the requested task is cognitively related, including components of memory, such as semantic memory (e.g. olfactory free-identification test, namely identification of odours without verbal labels choice). In fact, there are various pieces of evidence in the literature indicating that congenital blind present better performance than normal sighted individuals on tasks engaging verbal and auditory memory [33–35]. These overall findings are also in line with the studies conducted through evoked potentials, MRI olfactory bulb measurement and fMRI analysis [9, 12, 36].

In terms of sensory systems physiology, olfactory inputs are discreet and separated in time by sniffing, a critical and dynamic process of the olfactory environment. Indeed, selective attentional capture of olfactory stimuli is minimal, as well as is human olfactory awareness. On the contrary, visual and auditory inputs are nearly continuous in time, which means that these senses operate as attentional focus. Consequently, humans are suggested to be mostly “logged out” from their own abilities in olfaction [37]. In addition, according to a study focused on odour annoyance [38], people who notice odorants and are annoyed by them therefore think that they have a better sense of smell, yet in fact they do not. Actually, they simply pay more attention to smell. Thus, these aspects could explain why in some cases blind people outperformed normal sighted in olfactory and/or gustatory assessment; indeed, in the absence of a visual attentional spotlight, there is a higher selective attention focused on olfactory inputs.

Furthermore, sensory plasticity plays an important role in congenital blindness. Visual processing areas occupy almost one-third of the cortical surface. To congenitally blind people, this represents a large unoccupied cortical zone that can be used by other sensory systems [39]. For instance, visual input deprivation since birth has shown a relevant adaptive cross-modal plasticity in the human brain. This is because the visually deprived cortex is able to process information arising from other sensory modalities [40–42]. In addition, functional imaging studies showed that specific brain areas such as the right lateral orbitofrontal cortex mediates conscious olfactory perception [43], while the hippocampus is involved in memory [36]. These findings could unravel the neural compensatory mechanism of the blind’s increased odour awareness and memory [14]. Moreover, it could be meaningful to keep in mind perceptual variations: indeed, the way the brain is wired varies among individuals, because neural circuitry is shaped by the context and by previous experiences [44].

Thus, considering compensatory cross-modal plasticity, this perceptual setting could be even more complex in blind people. In other words, almost inconsistent results emerged by the literature, could be also partly reflecting the different pathways in which people’s brains are wired, imprinted by diverse sensory experiences over the course of life. As far as taste concerns, there are even less data, with only six studies, investigating gustatory *status* in blind participants, with or without olfactory assessment [23, 26, 28–31], so that it seems difficult to draw definitive findings. Indeed, Mahner and Freund results reflect methodological limitations of that time [29, 31], while Terner’s results were obtained in sighted patients with a digestive pathology where blind participants were the control group [23]. Regarding Smith and Gagnon studies on gustation, beyond the fact that the assessment methodologies were quite similar, results showed the absence of superior performance on blind participants [26, 28, 30].

On the other hand, pioneers like the psychologist Axel Rudolph, explored the idea of “shedding some light” on the sensory perception of the blind individuals (www.unsicht-bar-berlin.de/en/html/home_1.html). Unsicht-Bar is a small eatery run by the blind where clients enjoy wine, cuisine and conversation in pitch darkness. Following this idea, experimental psychologist Charles Spence addressed the topic by questioning whether turning off the lights really make the food and drink taste better in normal sighted subjects, and he showed that diners often found it difficult to distinguish between flavours in the absence of any visual cues [45, 46]. In fact, colour-taste and shape-taste crossmodal correspondences according to cultural context are well documented [47]. Regarding this topic, Weismann and collaborators investigated typical patterns of cortical activity by fMRI studies. They investigated whether cortical areas related to the olfactory and gustatory systems are also activated by eye closure without any other external stimulation. Results suggested that there are two different states of activity: with the eyes closed (an interoceptive state characterized by imagination and multisensory activity) and with the eyes open (an exteroceptive state characterized by attention and ocular motor activity) [48]. These interesting studies on normal sighted participants could support the idea that in blind individuals, the physiological mechanisms are extremely complex and unique so that they are not completely unravelled yet. Indeed, another interesting topic, but still poorly explored, is the ability to perceive food in blind people (sensory analysis). As far as we know, only two studies assessed this topic [31, 32], the first one by coffee powder tasting and the second one with a pure evaluation of smell performance in wine odour evaluation tasks. Interestingly in this latter work, what appeared different between early blind and sighted controls was not the smell performance but rather different internal categories constructing ability, probably due to the early vision deprivation (i.e. at birth or within the first few years of life). During “food flavour experience”, taste but also olfaction plays an important role, and it would be interesting to investigate the blind’ approach to the food, comparing normal sighted controls or a panel of trained people (e.g. as previously experienced with the “Tasting in the dark” event). Actually, the main limit of our preliminary study is that data were obtained on a small group of participants, in particular for the blind group. Anyway, it is meaningful that blind participants are distinguished from the other groups in wine tasting, which is surely a very complex sensory task and this datum, even though preliminary, is in line with the literature. It is worth mentioning that the food flavour is among the most complex and powerful of all human sensations, involving, besides smell and taste, vision, audition and somatosensory modalities [49, 50].

Furthermore, it is also important to consider an emerging interest in the chemical senses from Blind Unions. For example, in Italy a few years ago the Association “Vedere oltre onlus” from Rome, has planned a workshop for blind children (age range: 6–12 years) to experience foods by touch, olfaction and taste (http://vedereoltre.org/). In Sicily in 2009, the A.S.C.A. (Agenzia per la Sicurezza e il Controllo degli Alimenti, association established in 2005 by the “Italian General Department of Agriculture”) conducted a sensory analysis of the Sicilian agricultural products testing blind participants. Another experience regarding food products sensory analysis in blind participants was performed in Lucca in 2014 (https://2017.gonews.it/2014/10/27/percorsi-sensoriali-oltre-la-vista-dalla-santanna-un-progetto-in-collaborazione-con-lunione-italiana-ciechi-della-provincia-di-lucca/).

Summarizing, we think that a deeper analysis of the chemosensory abilities in blind individuals is necessary, particularly important, will be to consider the different populations of blind, congenital and acquired, and testing, through validated tests, a consistent number of individuals. As reported in the literature, the *status quo* for olfaction and taste in blindness is not consistent and detailed in all features when comparing touch and audition, even if there is a common popular opinion that blind outperform normal sighted in all sensory abilities. At the same time, it would be relevant to deeply evaluate the cognitive components, in particular memory, by means of different neuropsychological standardized tests. It could be possible that instead of sensory compensation, blind individuals rely on different cognitive strategies involving a better use of verbal memory processes. Thus, cognitive evaluation might provide important information on the role of memory in the smell and taste ability in blindness.

In addition, new scientific pieces of evidence on olfactory and gustatory abilities in blind people could bring out new possibilities to be enrolled in the food industry, as a food product tasting panel. For the coming perspective, this might represent an “added value” for food companies. Future research, including brain-imaging investigation, could help for a better understanding of multisensory systems integration and how is the brain reorganized when a perceptual source of information is impaired or temporarily unavailable.

## Data Availability

The data that support the findings of this study (“Tasting in the Dark” event, “Villa della Torre”, Verona, Italy) are available on request from the corresponding author [M.P.C.].
